# Hospital-Acquired Blood Stream Infection in an Adult Intensive Care Unit

**DOI:** 10.1155/2021/3652130

**Published:** 2021-07-03

**Authors:** Jeffrey Harte, Germander Soothill, John Glynn David Samuel, Laurence Sharifi, Mary White

**Affiliations:** Department of Critical Care Medicine, St Bartholomew's Hospital, Barts NHS Trust, London, UK

## Abstract

**Background:**

Hospital-acquired blood stream infections are a common and serious complication in critically ill patients.

**Methods:**

A retrospective case series was undertaken investigating the incidence and causes of bacteraemia in an adult intensive care unit with a high proportion of postoperative cardiothoracic surgical and oncology patients.

**Results:**

405 eligible patients were admitted to the intensive care unit over the course of nine months. 12 of these patients developed a unit-acquired blood stream infection. The average Acute Physiology And Chronic Health Evaluation II (APACHE II) score of patients who developed bacteraemia was greater than that of those who did not (19.8 versus 16.8, respectively). The risk of developing bacteraemia was associated with intubation and higher rates of invasive procedures. The mortality rate amongst the group of patients that developed bacteraemia was 33%; this is in contrast to the mortality rate in our unit as 27.2%. There was a higher proportion of Gram-negative bacteria isolated on blood cultures (9 out of 13 isolates) than in intensive care units reported in other studies.

**Conclusion:**

Critical-care patients are at risk of secondary bloodstream infection. This study highlights the importance of measures to reduce the risk of infection in the intensive-care setting, particularly in patients who have undergone invasive procedures.

## 1. Background

Hospital-acquired blood stream infections (BSIs) are a common and serious complication in critically ill patients. Nosocomial infection in intensive-care units (ICUs) has been shown to have prevalence as high as one-fifth of patients [1]. BSI occurs in approximately 7% of all admissions within their first month in the ICU [2]. Higher rate of infection in critically ill patients is associated with the use of central venous catheters, invasive ventilation, urinary catheters, and other invasive devices and equipment [3, 4]. These potentially preventable infections are associated with crude case fatality rates of approximately 40% [5] and increased rates of morbidity and length of ICU stay [6, 7].

Central-venous-catheter-related and ventilator-associated pneumonia (VAP) are the most common sources of secondary bacteraemia in critically ill patients [6]. Urinary-catheter-associated urinary tract infections are another secondary source. ICU-acquired BSIs are often Gram-positive pathogens such as coagulase-negative staphylococci and *Staphylococcus aureus*. *Escherichia coli* and enterococci are also commonly implicated in the development of bacteraemia in critically unwell patients [7, 8]. Recent studies during the COVID-19 pandemic have shown higher rates of Gram-negative infection in patients with COVID-19 requiring intensive care [9].

## 2. Methods

### 2.1. Setting

The study was conducted on a 16-bed adult mixed medical and surgical ICU in a tertiary oncology and cardiothoracic centre which can provide extracorporeal membrane oxygenation (ECMO), from April 1 to December 31, 2019. A review of these cases was undertaken due to a high rate of BSI reflected in the Intensive Care National Audit and Research Centre (ICNARC) quarterly report for this period. The observed rate of unit-acquired BSI per 1000 patient days was 4.4 compared to an expected rate of 1.8. Eligible patients were all patients admitted to the ICU over this period for a duration of 48 hours or more. The number of admissions to the ICU was 405, and the number of deaths were 111 (mortality rate 27.4%). Extracting the ICU-acquired bacteraemias and their related deaths from this dataset, the mortality rate is reduced to 27.2%.

### 2.2. Definitions

Definitions for ICU-acquired BSI were taken from the 2020 Centre for Disease Control (CDC) patient safety component manual [10]. A BSI was defined as the growth of a pathogen in a set of blood culture bottles. An ICU-acquired BSI was defined when the first bottle growth was more than 48 hours after admission to the ICU [7]. Positive bacterial culture for coagulase-negative *Staphylococcus* was excluded as it was felt likely to relate to contamination not to true bacteraemia.

A venous-catheter-related BSI was defined as a positive line tip culture in association with an ICU-acquired BSI [10]. A VAP was defined as pneumonia in patients mechanically ventilated for more than two days, who developed features of infection with positive culture on a sputum or bronchoalveolar lavage sample. A urinary-catheter-associated infection was defined as where an indwelling urinary catheter was in place for more than two days in a patient who developed features of infection with positive culture on a urine sample [10]. Suspected cases were defined by positive blood cultures and a strong clinical suspicion for bacterial source following clinical examination, e.g. increased purulent section load and signs of local infection around the catheter site.

Surgical admission was defined as any patient who had undergone a procedure admitted to the ICU in the immediate postoperative setting. Immunosuppression secondary to chemotherapy was defined as a patient having undergone chemotherapy within 30 days of their admission to the ICU.

## 3. Severity of Illness on Admission Was Defined Using the APACHE II Score

### 3.1. Data Analysis

Data collected by the ICNARC for our centre over the nine-month period were used to identify the ICU-acquired BSI cases. The cases were reviewed retrospectively for demographic, clinical, microbiological, and outcome data. Patient notes were analysed by a team made up of four clinicians who were not working in the unit during the period under review. Each case was reviewed by two members of the team independently. The following data were recorded for all patients: age, gender, date of admission, mode of admission, APACHE II score on admission, ICU length of stay, ICU outcome, purpose of admission, microbiological isolates, suspected sources of infection, device insertion and manipulation, and underlying chronic diseases. For descriptive analysis, numbers with percentages were used for categorical variables. Data were entered locally and calculated on the Microsoft Excel database. This was later merged to form a regional database for subsequent analysis.

## 4. Results

From April 1, 2019, to December 31, 2019, 405 patients admitted to the ICU were eligible for inclusion. Of those, 12 were diagnosed with a unit-acquired BSI giving an observed rate of 3.0%. The mean age was 63.8 years. The male-to-female ratio in the group of patients diagnosed with BSI was 1 : 1.

### 4.1. Risk Factors

Background- and admission-related risk factors for developing an ICU-acquired BSI are shown in Table 1. The mean ICU admission APACHE II score of patients who developed a unit-acquired BSI was 19.8, compared to 16.8 in the patients in the cohort not diagnosed with BSI. Three of the 12 admissions were surgical in nature, while the rest were composed of critically unwell oncological and cardiovascular or respiratory patients. The mean ICU stay of these patients was 28 days.

Of the BSI patients admitted to the ICU, five had a malignancy (mostly haematological) and were immunosuppressed having had a course of chemotherapy within 30 days of admission to the ICU. Of these, three were admitted with neutropenic sepsis, one was admitted following a cardiothoracic procedure, and the fifth patient was admitted with decompensated heart failure, as shown in Table 2. Two further patients were admitted due to postoperative cardiothoracic complications. Four patients were admitted with heart failure requiring inotropic support (including the previously mentioned patient on chemotherapy), and two patients were admitted for ECMO support for an exacerbation of chronic obstructive pulmonary disease and complete heart block, respectively. Five patients had been administered oral or intravenous steroids within seven days of their development of a BSI (two for chronic respiratory conditions, two for sepsis, and one empirically for worsening skin appearances).

All patients who went on to develop a BSI had some form of invasive equipment placed including central venous, arterial, and urinary catheters. High-risk equipment were considered to be pulmonary artery catheters or central venous lines; of our BSI cases, 67% had high-risk equipment placed. 83% were mechanically ventilated at some point during their admission. Five patients who developed a bacteraemia were treated with intravenous steroids. Four of the 12 cases (33%) died.

### 4.2. Timing

**Figure 1:**
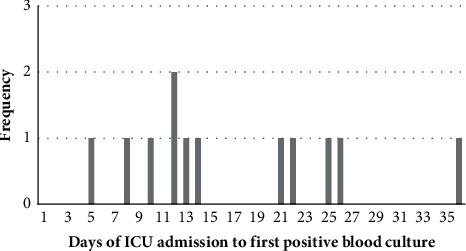
Histogram showing the timing of the day of admission to the intensive care unit when the first positive blood culture occurred in patients with a unit-acquired blood stream infection.

There was a range of early and late infections. One patient developed a bacteraemia within the first seven days, six cases subsequently were diagnosed between seven and fourteen days, and five cases were diagnosed in the following twenty days (Figure 1).

## 5. Microbiology

**Figure 2:**
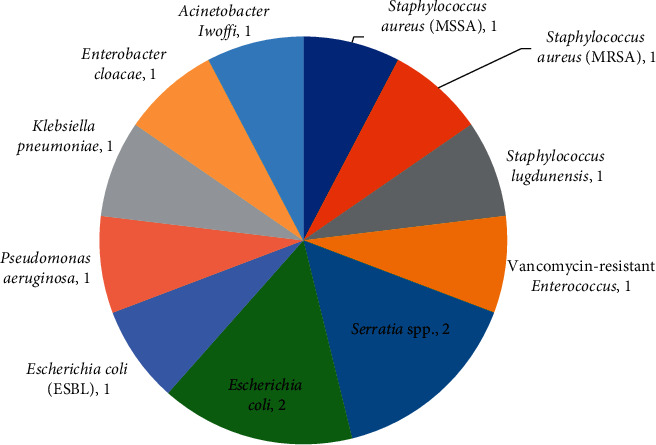
Pie-chart showing the distribution of pathogenic isolates for patients identified as having a unit-acquired bloodstream infection.

13 pathogenic isolates were grown on blood culture in total (Figure 2). *Escherichia coli (E. coli)* was the most common causative pathogen (three cases), with one of these cases being an extended spectrum beta lactamase- (ESBL-) producing organism. There were two cases of *Serratia* and two cases of *Staphylococcus aureus* (one methicillin-sensitive *Staphylococcus aureus* (MSSA) and one methicillin-resistant *Staphylococcus aureus* (MRSA)). One patient grew an *Enterococcus* and *E. coli* simultaneously. Three cases were multidrug-resistant organisms: ESBL *E. coli*, MRSA, and vancomycin-resistant *Enterococcus* (VRE). Nine of the 13 cases were Gram-negative microorganisms. It should be noted that all patients on arriving in our ICU were screened for MRSA. However, it is unclear from the documentation whether the patient who developed an MRSA bacteraemia was decolonised on identification of a positive MRSA swab twenty-five days prior to their first positive blood culture. The case of the VRE BSI did not have screening for VRE.

### 5.1. Source of Infection

**Figure 3:**
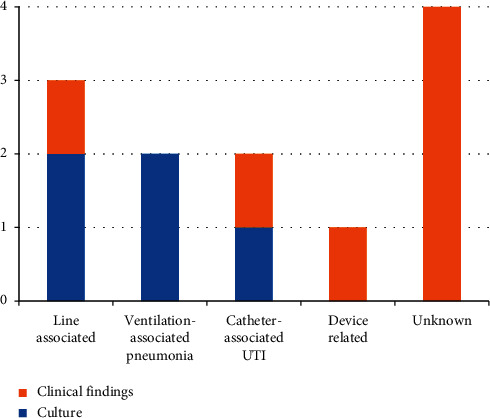
Graph showing the suspected source of blood stream infection based on clinical findings and culture results for the patients with unit-acquired bloodstream infection.

For the patients with an ICU-acquired BSI, a focus of infection was identified with growth of the same pathogen on other culture samples (sputum, urine, or line tip) in five cases. The most commonly confirmed sources of BSI were VAP and line associated with two cases of each. There was a single case of urinary-catheter urinary tract infection- (UTI-) associated BSI. Seven cases of BSI did not grow the same pathogen on any other culture sample. However, based on clinical findings, one case of BSI was suspected to be urinary catheter acquired, one was identified as being due to a line infection, and one was felt to be secondary to an infected pacemaker device (Figure 3). Following review of the clinical cases and culture results, the suspected source of BSI remained unclear for four cases.

## 6. Discussion

The range of pathogens causing BSI in this cohort of patients is consistent with that seen in other intensive care unit studies. This research demonstrated a higher incidence of Gram-negative pathogens causing bacteraemia compared to larger scale studies, which have shown a predominance of Gram-positive organisms [2, 8]. Postoperative BSIs are most commonly attributed to Gram-positive organisms, and this has been demonstrated in multiple previous studies [11]. The patients admitted to our unit would be considered more complex than those admitted to a general ICU in a tertiary or district general hospital as they have been referred from these centres for specialist care. Immunocompromised patients do have higher rates of Gram-negative bacteraemias due to prolonged periods of neutropaenia and chemotherapy-induced gastrointestinal mucositis [12, 13]. As immunocompromised patients did not make up the majority of the patients admitted to this ICU, this, whilst likely a contributing factor, does not fully explain the results.

During the COVID-19 pandemic, it has been noted that the rate of Gram-negative BSI in patients with COVID-19 infection requiring ICU care has been high [14]. This study provides useful baseline data of the rates of BSI in an ICU prior to the pandemic.

Line-associated bacteraemia was the most common cause of secondary BSI (one based on clinical findings and two based on cultures), followed by VAP and urinary-catheter-associated infections. In the cases of line-associated infection, the notes were reviewed to verify if lines had been inserted using an aseptic technique. In two of the three cases, the documentation surrounding the venous catheter insertion was unsatisfactory. This should include clear documentation on timing, use of maximal sterile precautions, US guidance, site of insertion, use of skin drapes where possible, and number of passes. All these features have been shown to reduce complications [15]. This has changed since completion of this audit, and there is now a preconfigured document on our computer system that must be completed at the time of central line insertion. This research will reinforce the importance of this.

For the patients found to have BSI secondary to VAP, documentation of VAP care bundle use was reviewed. In both cases of BSI secondary to VAP, there was inadequate documentation of the VAP care bundle. There is strong evidence to suggest the VAP care bundles have been shown to improve clinical outcomes and appear feasible and safe. Simple tasks including elevating the head of the bed ≥30°, good oral care, regular suctioning, and good hand hygiene all contribute to reduced rates of VAP [16]. As such, these should be a standard of care on the ICU. Inadequate documentation of VAP care bundles may have been due to the fact that these study data were captured during a transitional period when nursing staff were moving from paper to electronic documentation for all notes. Observations, fluid balance, and drugs are recorded on A2 sheets of paper; nursing documentation and other recordings are completed using electronic documentation. Care bundles, however, are often still recorded in paper nursing notes which are to be later transcribed to the computer system. This process was not adequately recorded in our two VAP cases, and paper nursing documentation was not accessible for this study. This research locally has highlighted the importance of this transcription in managing and recording interventions, and nurse-led teaching with regards to this has been ongoing.

All patients were screened for MRSA on admission to the ICU. It was not clear from the documentation whether the patient who acquired the MRSA bacteraemia had received appropriate eradication therapy. The patient who developed VRE bacteraemia did not have VRE screening. In patients with persistent pyrexia of unknown origin, all patients had devices or lines removed or replaced where possible.

BSI is an important cause of morbidity and mortality in the ICU. This is reflected in the mortality rate of 33% in this patient cohort. The importance of thorough clinical examination and septic screen testing to identify a source of infection to guide targeted antimicrobial therapy is key. Patients in the ICU are at high risk of secondary infection. This study highlights the importance of an aseptic technique during line and catheter insertion, MRSA decolonisation, VAP care bundles, and the recorded documentation of these.

## 7. Study Limitations

The authors recognise the exclusion of coagulase-negative *Staphylococcus* blood cultures may have underestimated the true rate of unit-acquired BSI. In addition to this, as per our definition for a unit-acquired bacteraemia, any individuals who had an ICU admission of less than 48 hours but developed a subsequent bacteraemia once stepped down to the ward would not have been captured in our dataset. This may also serve to underestimate the rates of BSI on the unit.

The case reviews would have been enhanced by access to paper nursing documentation to assess the quality of the documentation for the VAP care bundles and MRSA decolonisation. In four cases of BSI, no cause of infection was identified. Review of the notes in these cases did not reveal any further information as to the cause of the bacteraemia.

## 8. Conclusions

This study demonstrates a higher proportion of Gram-negative bacteraemia compared to large-scale ICU studies in the literature where Gram-positive pathogens predominated. This may be attributed to our cohort of patients, many of whom were immunosuppressed or had complex comorbidities requiring transfers to a specialist centre in the first instance. Our mortality rate for patients with BSI did not differ from rates seen in other intensive care units.

Critical-care patients are at high risk of infection, and the importance of measures to reduce the risk of secondary infection through comprehensive ICU care including line insertion, VAP care bundles, and documentation remains high. The importance of MRSA screening and decolonisation has been highlighted in this research for all patients being admitted to our ICU.

There has been increased reporting of Gram-negative bacteraemias in COVID-19, and this research provides valuable data on ICU-acquired BSIs in the nine months prior to the outbreak of the COVID-19 pandemic.

## Figures and Tables

**Table 1:** Patient factors considered to be associated with developing a bloodstream infection on the intensive care unit.

Factor	Number (%) of BSI cases
Mechanically ventilated	10 (83)
High-risk equipment/Procedures	8 (67)
Immunosuppression	5 (42)
Malignancy	5 (42)
Steroids	5 (42)
Surgical admission	3 (25)
Diabetes mellitus	2 (17)
Multiple line changes over the course of patients stay	2 (17)
Chronic kidney disease	2 (17)
Chronic obstructive pulmonary disease	1 (8)
Liver disease	1 (8)

**Table 2:** Reasons for admission to the intensive care unit for patients who developed a unit-acquired blood stream infection.

Reason for admission to the intensive care unit	Number of cases (%)
Decompensated heart failure	4 (33)
Postoperative complications	3 (25)
Neutropaenic sepsis	3 (25)
Complete heart block	1 (8)
Infective exacerbation of chronic obstructive pulmonary disease	1 (8)

## Data Availability

Excel sheets with which data were collected on and calculations were made can be provided on request.

## Abbreviations

APACHE II: Acute physiology and chronic health evaluation II
BSI: Blood stream infection
ICU: Intensive care units
VAP: Ventilator-associated pneumonia
COVID-19: Coronavirus disease 2019
ECMO: Extracorporeal membrane oxygenation
ICNARC: Intensive Care National Audit and Research Centre
CDC: Centre for Disease Control
*E. coli*: *Escherichia coli*
ESBL: Extended-spectrum beta lactamase
MSSA: Methicillin-sensitive *Staphylococcus aureus*
MRSA: Methicillin-resistant *Staphylococcus aureus*
VRE: Vancomycin-resistant *Enterococcus*
UTI: Urinary tract infection.
